# Supplementary figures and images for: Unveiling the impact of organically activated biochar on physiological, biochemical, and yield attributes of maize under varied field moisture conditions
Source: PeerJ. 2024 Oct 1;12:e17883. doi: 10.7717/peerj.17883 (PMC11451447; doi:10.7717/peerj.17883)

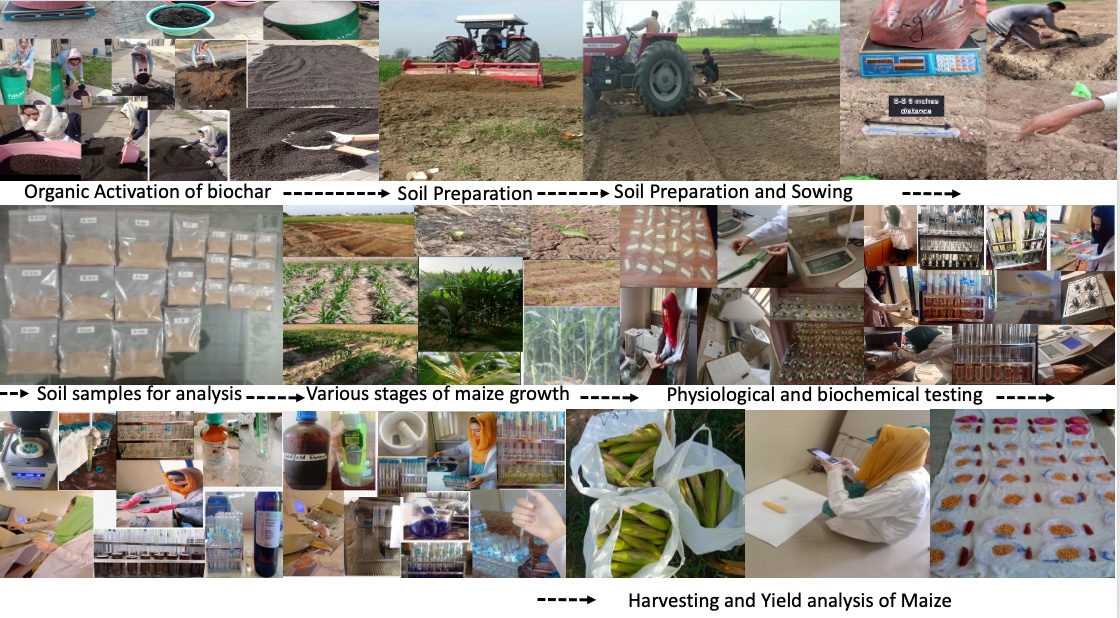

Supplement: Supplemental Information 3 [file peerj-12-17883-s003.jpg]
