# Supplementary material for: Unveiling the impact of organically activated biochar on physiological, biochemical, and yield attributes of maize under varied field moisture conditions
Source: PeerJ. 2024 Oct 1;12:e17883. doi: 10.7717/peerj.17883 (PMC11451447; doi:10.7717/peerj.17883)
Supplement: Supplemental Information 4 [file peerj-12-17883-s004.docx]

#create a chord diagram but without labeling

chordDiagram(HI_data, grid.col = col, annotationTrack = "grid", preAllocateTracks = 1)

#add the labels and axis

circos.trackPlotRegion(track.index = 2, panel.fun = function(x, y) {

xlim = get.cell.meta.data("xlim")

ylim = get.cell.meta.data("ylim")

sector.name = get.cell.meta.data("sector.index")

#print labels

circos.text(mean(xlim), ylim[1] + 2.5, sector.name,

facing = "clockwise", niceFacing = TRUE, adj = c(0, 0.5), cex=0.6)

#print axis

circos.axis(h = "top", labels.cex = 0.5, major.tick.percentage = 0.2,

sector.index = sector.name, track.index = 2)

}, bg.border = NA)

#saving the plot (high definition)

dev.copy(jpeg,'plot.png', width=8, height=8, units="in", res=500)

dev.off()

#2

install.packages("gplots")

library(pheatmap)

ma <- as.matrix(HI_data[, 2:27]) # convert to matrix

rownames(ma) <- c("0 t/ac AB+100% IW+V1","0 t/ac AB+70% IW+V1","0 t/ac AB+50% IW+V1","2 t/ac AB+100% IW+V1", "2 t/ac AB+70% IW+V1", "2 t/ac AB+50% IW+V1", "4 t/ac AB+100% IW+V1", "4 t/ac AB+70% IW+V1", "4 t/ac AB+50% IW+V1", "0 t/ac AB+100% IW+V2", "0 t/ac AB+70% IW+V2", "0 t/ac AB+50% IW+V2", "2 t/ac AB+100% IW+V2", "2 t/ac AB+70% IW+V2","2 t/ac AB+50% IW+V2", "4 t/ac AB+100% IW+V2", "4 t/ac AB+70% IW+V2", "4 t/ac AB+50% IW+V2","0 t/ac AB+100% IW+V3","0 t/ac AB+70% IW+V3","0 t/ac AB+50% IW+V3","2 t/ac AB+100% IW+V3", "2 t/ac AB+70% IW+V3", "2 t/ac AB+50% IW+V3", "4 t/ac AB+100% IW+V3", "4 t/ac AB+70% IW+V3", "4 t/ac AB+50% IW+V3")

pheatmap(ma,

scale = "column",

clustering_method = "average", # average linkage

drop = FALSE, # the 1st column as color bar

show_rownames = TRUE

)
